# Complex Anterior Supralevator Anal Fistula With Prostatic Abscess Treated With Ksharasutra: A Case Report

**DOI:** 10.1155/2024/6019946

**Published:** 2024-10-08

**Authors:** Punchoor Ramesh Bhat, Vipin T. A.

**Affiliations:** Acharya Sushrutha Healthcare Pvt. Ltd, ITI Layout, Bengaluru 560056, India

**Keywords:** anal fistula, Ksharasutra, sonofistulogram, supralevator

## Abstract

Anorectal diseases are a major health threat in the field of health sciences. Fistula-in-ano is one of the treatable complex benign lesions of the rectum and anal canal. Complex high anal fistulas can reoccur even after surgical treatment. Establishing a cure for cryptoglandular fistula-in-ano is problematic, as a significant percentage of these diseases persist or recur if the internal opening of the anal fistula is left untreated. Here, we report a case of complex left anterolateral supralevator anal fistula with communication to the prostate gland that forms a prostatic abscess, as it is very rare. After following *Ksharasutra* (Ayurvedic medicated seton) treatment with regular wound care, significant regression in the condition was observed. A follow-up scan showed no evidence of fistula-in-ano. A 50-year-old businessman presented with complaints of discomfort deep inside the rectum and perineum associated with pain at the base of the scrotum and pus discharge from the perianal region for 1 year. He was diagnosed to have a complex left anterolateral supralevator anal fistula with communication to the prostate substance after a thorough clinical examination and transrectal ultrasonography. After undergoing Ksharasutra treatment for 4 months, pus discharge completely stopped, and sonofistulogram report showed no evidence of fistula-in-ano. Images of the sonofistulogram report were documented before and after the treatment. This article highlights the unique feature of Ksharasutra therapy where the fistula extending to the prostate was treated with no loss of function of the anal sphincter.

## 1. Introduction

Fistulae-in-ano form a good majority of treatable benign lesions of the rectum and anal canal. More than 90% of these cases result from cryptoglandular infections [1]. *Acharya Sushruta*, an Ancient Indian surgeon, has described blind fistulas with either internal or external openings as Bhagandara in *Sushruta Samhita* [2]. *Ayurveda* is well-known for treating *Bhagandara* with *Ksharasutra* application, which is now an accepted modality of treatment everywhere after thorough research. Ksharasutra (medicated seton) is being practiced in India with a high success rate (recurrence of 3.33%) in the management of complicated anal fistula [3]. The case reported here showed complete remission of complex anal fistula communicating to the prostate gland after treatment by Ksharasutra.

## 2. Case Presentation

A 50-year-old businessman presented with complaints of discomfort deep inside the rectum and perineum associated with pain at the base of the scrotum and pus discharge from the perianal region along with mild feverish feeling since 1 year. The patient had a previous history of repeated urinary tract infections treated with antibiotics prescribed by a family physician.

## 3. Clinical Findings

Perianal examination revealed an induration in the left anterolateral region of the anus nearer to the base of the scrotum at 1 o'clock position (one external opening). Digital rectal examination revealed induration and tenderness of the anal mucosa at the 1 o'clock position and 2 o'clock position at the level of the anal valve. Perianal skin exfoliation, edema, and chronic skin inflammation were observed. Active discharge through the external opening was observed.

## 4. Diagnostic Assessment

**Figure 1:**
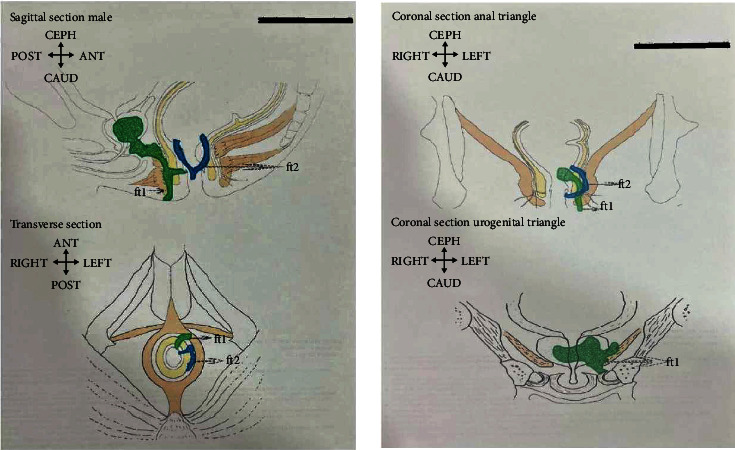
Image of the diagrammatic representation of sonofistulogram before Ksharasutra treatment.

Sonofistulogram done by endoanal and perianal ultrasound probes revealed two fistulous tracks. Track 1 was located at the left anterolateral aspect of the anal canal with an internal opening at 1 o'clock position at the level of the anal valve extending above the levator ani and passing through the apex of the prostate forming an abscess in the substance of prostate. Track 2 was located at the left side of the anal canal with an internal opening at 2 o'clock position below the level of the anal valve communicating to the rectum (Figure 1).

Diagnosis of the left anterolateral supralevator anal fistulae with prostatic abscess was made.

## 5. Treatment and Outcome

The standard technique of preparation of Ksharasutra, type of Ksharasutra, method of threading, and change of thread were followed as per the standard protocol of Banaras Hindu University, Varanasi, India [3].

**Figure 2:**
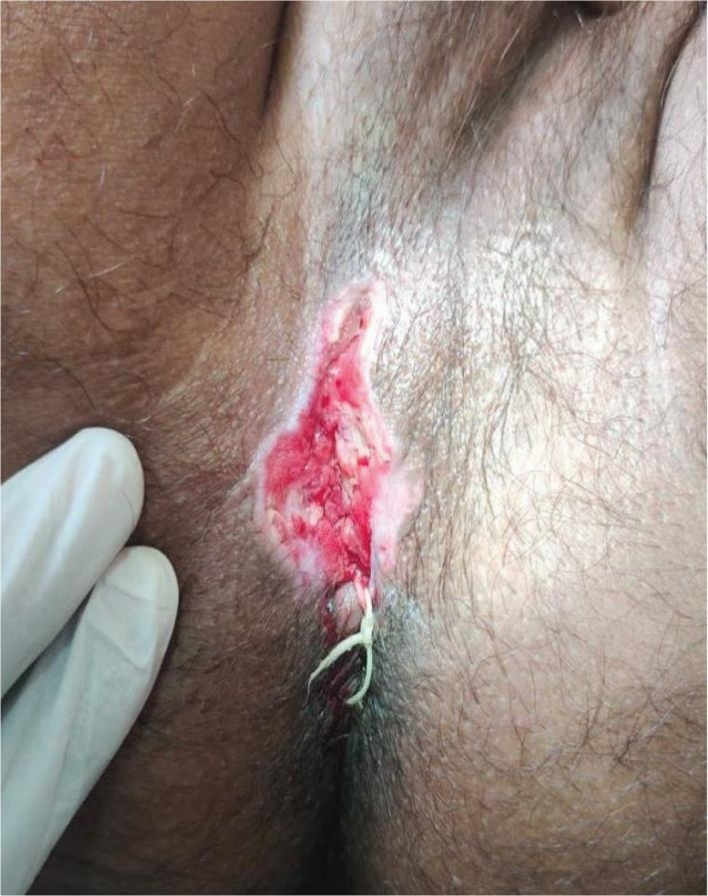
Image of the perianal region during treatment with Ksharasutra in situ.

A window was created for track 1 at 1 o'clock position at the perianal region, 1.5 cm away from the anal verge, by splitting the tract and seton (Barbour thread no. 20) placed under spinal anesthesia. Ksharasutra was changed after 13 days of the initial procedure (Figure 2). Every week, the Ksharasutra change was continued for 11 weeks. At the end of the 11^th^ week, the Ksharasutra cut the tract completely leaving a small skin wound. At the end of the 12^th^ week, the skin wound also completely healed. *Triphala guggulu* and *gandhaka rasayana* tablets, which are Ayurveda polyherbal compound medications, were given orally throughout treatment at 2 BID00 each. The patient had one course of levofloxacin 500 mg once daily from the 7^th^ day of treatment for 1 week as per the physician's advice. Patient had paracetamol 650 mg on an SOS basis for pain.

Track two was curetted and seton placed. The procedure of the change of Ksharasutra was done weekly just as in track 1. It healed completely at the end of 3^rd^ week.

After treatment and wound healing, the patient was relieved of pain and pus discharge. A follow-up sonofistulogram revealed no evidence of fistula-in-ano, except a small residual cavity in the region of the prostatic abscess. After a complete healing follow-up of 2 years and 7 months, no signs of recurrence of the fistula or any symptoms of the urinary tract were noticed which suggests residual abscess cavity of the prostate might have fibrosed eventually.

Digital rectal examination revealed adequate tonicity of the sphincter.

## 6. Discussion

Ksharasutra treatment for anal fistula has been mentioned in *Sushruta Samhita*, an ancient Indian surgical textbook [4].

The rate of disease recurrence in Ksharasutra therapy is 3.33% [3]; in conventional surgery, it is ~60% [5]. Ksharasutra therapy has shown a significant success rate reported as 96.5% with a low incidence of incontinence [6]; in other studies, it is from 94.1% to 100% in single-center prospective studies to 96% in multicenter randomized controlled trials involving both low and high types of fistulas with no or minor incontinence [7–9]. Anal continence is significantly affected after anal fistula surgery, mainly due to sphincteric lesions that affect anal canal pressures and changes in morphological and functional parameters [10]. Outcomes of fistulectomy and even of cutting seton have also not been satisfactory in terms of incontinence rates reaching up to 25% for the former [11] and 67% for the latter [12].

Three hundred patients with transphincteric and intersphincteric complex fistula and (130 cases of recurrent fistula) underwent the IFTAK technique (interception of fistula track with application of Ksharasutra). Successful healing was achieved in 280 (93.33%) in 6–8 weeks. 3.67% of the cases reported mild impairment of continence on the Wexner incontinence scoring system. It also showed that Ksharasutra, besides eradicating the infected crypt and facilitating drainage, gradually lays open the proximal track. In contrast, the distal track heals spontaneously, being cutoff from the primary source of infection demonstrated by a transrectal scan and histopathological examination [13].

A study conducted on two types of Ksharasutra versus cutting thread on 300 patients showed significant granulation, early fibroblastic proliferation, absence of microabscess, and effective debridement of necrotic tissue on the HPE study of the fistula tract. Faster healing, adequate patency of wound facilitating sufficient drainage, a good antimicrobial environment in the tract, was noticed in the Ksharasutra group when compared to plain thread/cutting seton. Recurrence was 12% in the plain thread group, and in Ksharasutra group, it was 1% [14]. However, further comparative studies on Ksharasutra in the management of anal fistula are useful.

The presentation of anal fistula in clinical practice varies and sometimes is difficult to diagnose. Some unusual anal fistulae reported earlier are fistula communicating to the foot [15] and thigh [16]. The author also treated a patient who had an anterior anal fistula and had earlier undergone surgery 26 times in the United States, which was not successful. He was treated with Ksharasutra successfully without recurrence 5 years ago [17]. We also have experience in treating anal fistula communicating to the thigh, scrotum, deep gluteus, hip, supralevator, and para rectum areas using medicated seton successfully in the last two decades. In all these cases, distal communications of the fistula track were obliterated during the follow-up period. Here, in this case, the tract coursing toward the pelvis and reaching the prostate gland forming an abscess was also detected and obliterated; the patient did not show any symptoms related to the prostate or urinary tract when followed up for 2 years. This may be a rare case that is of clinical importance for practicing colorectal surgeons.

In this case, two different fistulae with deep extensions to the prostate gland and rectum communication were found. It is not easy to do either fistulotomy or fistulectomy in this case as it was extending to the deeper plane. To save the sphincter and also to avoid dissection, this method was followed. The author also had experience of treating such complex anal fistula and regularly follows this technique which yields good outcomes without recurrence.

The major challenges and key factors involved are as follows:

a. Finding an appropriate site to create a window to divert secretions and to achieve adequate drainage which helps in healing anal fistula without recurrence.
b. To place a seton involving the infected crypt through the internal opening. It is a precise and accurate surgical procedure. The surgeon should have a correct understanding of the course of the fistula which is different in each fistula. Surgeons should craft procedures depending on the spread and branches of the fistula.

This principle is generally followed in Ksharasutra therapy for anal fistula with long distal extensions [15]. The small tract was treated with Ksharasutra, involving an internal opening and not the entire tract. Ksharasutra was changed with minimal pain as an outpatient department procedure while the patient was doing his routine activities.

## 7. Conclusion

Ksharasutra therapy has a high success rate compared to other surgical procedures in the cases of complex fistula-in-ano. It can be followed as the first line of treatment for complex anal fistula. Ksharasutra is very useful in treating the secondary extension of complex fistulas while preserving the function of the anal sphincter. However, the protocol followed in this case throws light on further studies, and it will be useful to get more insight into this technique for practicing surgeons.

## Data Availability

Data will be made available on request.
